# The Effect of Midlife Cardiovascular Risk Factors on Cognitive Function Nearly Half a Decade Later

**DOI:** 10.1093/geroni/igaa057.3297

**Published:** 2020-12-16

**Authors:** Teresa Warren, Shandell Pahlen, Hong Xian, Jennifer De Anda, William Kremen, Carol Franz

**Affiliations:** 1 San Diego State University, La Jolla, California, United States; 2 University of California, Riverside, Riverside, California, United States; 3 Saint Louis University, St. Louis, Missouri, United States; 4 University of California San Diego, San Diego, California, United States

## Abstract

Several cardiovascular risk factors (CVRFs) have been associated with poor cognitive function. However, few studies have examined these factors longitudinally during midlife. We hypothesized that more midlife CVRFs would predict worse cognitive function approximately six years later. Participants were 886 men who participated in waves 2 and 3 of the Vietnam Era Twin Study of Aging. The American Heart Association’s “Life’s Simple 7” index was used to measure CVRFs. CVRFs were assessed at mean age 61 (range 55-66) and included smoking, physical activity, diet, body mass index, cholesterol, glucose, and blood pressure. Each factor was coded on a 3-point scale (0-2), ranging from poor to ideal status. These scores were then used to create a composite CVRF index (0-14). We examined several cognitive domains assessed at mean age 67 (range 61-73): abstract reasoning, episodic memory, processing speed, executive function, working memory, general verbal fluency, and semantic fluency. Analyses were adjusted for ethnicity, and education, and mean age 61. In the generalized estimating equation models, there were significant main effects indicating that the CVRF index at mean age 61 significantly predicted cognitive function at mean age 67 in episodic memory, 95% CI [.01, .08], p = .01, processing speed, 95% CI [.02, .09], p = .01, and executive function, 95% CI [.00, .06] ], p = .03. The CVRF index did not predict cognitive function in the other cognitive domains. These results suggest that poor cardiovascular health in late midlife may exacerbate cognitive decline.

